# The levels of CD16/Fc gamma receptor III A on CD14^+^CD16^+^ monocytes are higher in children with severe *Plasmodium falciparum* anemia than in children with cerebral or uncomplicated malaria

**DOI:** 10.1186/1475-2875-9-S2-P29

**Published:** 2010-10-20

**Authors:** Lilian A Ogonda, Alloys SS Orago, Michael F Otieno, Christine Adhiambo, Walter Otieno, José A Stoute

**Affiliations:** 1 Department of Pre-clinical sciences, Kenyatta University, Nairobi, Kenya; 2 US Army Medical Research Unit and the Kenya Medical Research Institute, Nairobi, Kenya; 3 Department of Medicine, Division of Infectious Diseases, and Department of Microbiology and Immunology, the Pennsylvania State University College of Medicine, Hershey, PA, USA

## Background

Fc gamma receptor IIIA (CD16/FCγRIIIA) on monocytes/macro-phages may play an important role in the pathogenesis of severe malarial anemia (SMA) by promoting phagocytosis of IgG-coated uninfected red cells and by allowing the production of tumor necrosis factor alpha (TNF-α) upon cross-linking by immune complexes (ICs). However, not much is known about the differential expression of this receptor on monocytes of children with severe malaria and uncomplicated malaria.

## Materials and methods

We investigated the expression of CD16/FCγRIIIA on monocytes of children with SMA, cerebral malaria (CM), and their age-matched uncomplicated malaria controls by flow cytometry. Since CD14low (CD14+) monocytes are considered more mature and macrophage-like than CD14high (CD14++) monocytes, we also compared the level of expression of CD16/FCγRIIIA according to the CD14 level and studied the relationship between CD16/FCγRIIIA expression and intracellular TNF- production upon stimulation by ICs.

## Results

CD16/FcγRIIIA expression was the highest overall on CD14^+^CD16^+^ monocytes of children with SMA at enrollment. At convalescence, SMA children were the only ones to show a significant decline in the same parameter. In contrast, there were no significant differences among groups in the expression of CD16/FCγRIIIA on CD14^++^CD16^+^ monocytes. A greater percentage of CD14^+^CD16^+^ monocytes produced TNF-α upon stimulation than any other monocyte subset, and the amount of intracellular TNF-α correlated positively with CD16/FcRIIIA expression. Furthermore, there was an inverse correlation between hemoglobin levels and CD16/FCγRIIIA expression in children with SMA and their controls.

## Conclusions

These data suggest that monocytes of children with SMA respond differently to *Plasmodium falciparum* infection by overexpressing CD16/FCγRIIIA as they mature, which could enhance erythrophagocytosis and TNF production.
